# Lifestyle Medicine: Why Do We Need It?

**DOI:** 10.1007/s40670-018-00632-x

**Published:** 2018-11-14

**Authors:** Hanno Pijl

**Affiliations:** Dept of Internal Medicine, Section of Endocrinology, Leiden University Medical Center, Leiden, Netherlands

**Keywords:** Healthcare system, Non-communicable disease, Lifestyle medicine

## Abstract

Modern medicine has its roots in the nineteenth century, when bacteria and viruses were increasingly recognized as the primary cause of the most prevalent diseases of that era. In the early twentieth century, the discovery of antibiotics provided a cure for infectious disease (Aminov, Front Microbiol 1:134, 2010). Moreover, the advent of effective anesthesia allowed more extensive surgery to manage the damage done by accidents. When we got sick, we attended a medical doctor, who prescribed a pill or performed surgery, which essentially cured our illness. This health care model worked perfectly well and still does in case of infectious disease or fractures. However, the nature of contemporary disease has changed profoundly over the last century, and we failed to appropriately adapt our health care system.

Today’s world is plagued by a tsunami of chronic, “age-associated,” non-communicable disease. In fact, we are so used to sickness in old age that we came to believe that it is an inevitable consequence of the aging process. However, there is no scientific basis for such belief. People can maintain good health well into old age [2, 3]. So why is there mass illness in the world today?

Aging comes with cell damage. For example, in every cell of our body, DNA gets damaged some 10^4^ times [4]. The damage is brought about by spontaneous mutations, by-products of metabolism and environmental elements like toxins, X-rays, and ultraviolet radiation. Intricate repair mechanisms continuously restore genomic integrity, but some damage inevitably escapes restoration on a regular basis. In the course of life, persistent damage accumulates, which induces inflammation and functional decline [5].

The damage accumulation rate is the resultant of the rate of induction on the one hand and efficacy of repair on the other. Therefore, the more extensive the exposure to noxious stimuli, the quicker damage accumulates and disease develops. Moreover, molecular repair systems (like every other biosystem in animals) are made up of what we eat. Thus, nutrient deficiencies can compromise the structure and functional capacity of the (DNA) damage response, which accelerates damage accumulation [6].

The vast majority of chronic non-communicable diseases is caused by gene-environment interactions. Behavioral and environmental cues trigger illness, the nature of which is determined by our (epi-)genetic architecture [7]. Too much of the wrong food, prolonged stress, lack of physical exercise, bad sleep, smoking, and toxins are the most important “exogenous” triggers. Indeed, the environment is paramount in the pathogenesis of chronic disease [8].

Almost always multiple cues are involved in the etiology of any one non-communicable disorder, but the primary culprit may differ among distinct illnesses and individuals. Without exception, chronic disease is marked by systems failure: a multitude of physiological checkpoints go haywire [9]. The chronic disorders of today are multifactorial systems diseases, in contrast to the acute, unifactorial, relatively simple infectious diseases and accidents we were faced with in the nineteenth century. And yet, we still apply the model of our health care system of those days. Perhaps the tremendous success of antibiotics has lured us into the illusion that we can solve any disease with chemicals. But this is difficult to envision with regard to multifactorial systems diseases for at least two reasons.

Since multiple physiological processes go awry simultaneously in systems disease, and drugs generally target only one specific process, a multitude of drugs is necessary to restore homeostasis (if at all possible with drugs). Many of my patients with type 2 diabetes come into my office using a host of pills to control blood glucose, blood pressure, cholesterol, and the consequences of diabetic complications. All of these drugs have side effects. Moreover, no one knows how the different drugs interact in the context of the physiology of my patient. And last, but not least, drugs are costly.

But there is another, even more important reason why it is illusory that drugs will solve the problems. They do not address the root cause of any chronic disease: the way we live our life. Basically, we fight a running battle when we treat non-communicable disease with drugs without addressing lifestyle. Lifestyle modification redresses the fundamental drivers of the (cellular) damage-causing disease. Type 2 diabetes has long been considered a chronic, progressive, incurable disease [10], until it was clearly shown that calorie restriction can essentially cure it [11]. This is exactly the reason why we need lifestyle medicine: without it, we are trying to empty the ocean with a thimble.

Lifestyle medicine is *not* an alternative to traditional medicine. It is a necessary adjunct. It needs to be integrated with our formerly fruitful, but old-fashioned, traditional approach to disease. Our current *health* care system actually takes care of the sick. It is probably easier to prevent than to cure most non-communicable diseases. We should take better care of our health. However, when people get sick nonetheless (and some of us certainly will because of stochastic damage accumulation), lifestyle adaptation needs to be primary treatment. If appropriate lifestyle change does not (completely) restore health, drugs are there to support the healing process.

The integration of lifestyle medicine with our traditional approach requires a lot from our health care system. First of all, sick people should take center stage in their treatment. People should learn to feel responsibility for their own health. We now expect a doctor to take that responsibility. This is a blind alley in the context of lifestyle medicine. After all, no one but the patient him- or herself can change ingrained habits. The medical doctor of the future is merely an advisor.

People can only take center stage if they know what their disease is all about. Here lies the most important role of future medical doctors: they have to inform their patients about the origins of their disease and the possibilities to restore their health. The patient then decides which way to go. I often hear objections, doubting the capacity of people to take that responsibility. However, I do believe that this has much to do with culture and education. If we teach our kids early on that they, and only they, have to take care of their own health, it will be much easier for people to take the lead in case of illness. It is too ridiculous for words that we teach our children math, but fail to tell them about their health.

The diagnostic process needs to include the socio-economic, mental, spiritual, and behavioral aspects of a patient’s life in addition to biological markers of health and disease. Indeed, it is virtually impossible to achieve meaningful lifestyle change in the context of deleveraging and fixation on financial debts, for example. As the psycho-social conditions of people critically impact on their behavior, the treatment of chronic disease should address relevant issues in this domain as well.

Although medical doctors will be paramount to future health care (informing, advising, and checking patients), they will probably not be the ones who coach patients’ journeys towards health. Specialized, paramedic health coaches will probably do that job. It requires in-depth knowledge of the disease at hand, as well as expertise in behavior change strategies. Notably, the fact that patients must be in the lead with regard to their health does *not* mean that they should be abandoned to their fate. It is very difficult indeed to live a healthy life in the context of the current society.

These requirements of future (lifestyle) medicine obviously bear upon medical education programs. We need to teach doctors to take a step back and be an advisor rather than a leader. Doctors will have to know about societal structures, cultural distinctions, religious aspects of behavior, and the ins and outs of behavior change, in addition to detailed knowledge of gene-environment interactions in the etiology of (chronic) disease. Education should also include an extensive training in motivational communication. And finally, we may need to include a course in philosophy of science, particularly to address the issue of scientific credibility of lifestyle medicine. Indeed, we have to recognize that the methods of gathering scientific evidence for the efficacy of lifestyle interventions are not as straightforward as the collection of data to document the effects of drugs. After all, there is no such thing as a “placebo lifestyle intervention.” Moreover, changing one aspect of lifestyle often impacts on other aspects as well. Novel, practice-based scientific methods are required to meet the needs of proper substantiation of lifestyle medicine.

The current epidemic of chronic non-communicable disease requires a modern approach of patients and health. Although we managed to control infectious disease by using chemicals (albeit not in every part of the world!), this strategy will not suffice to similarly control non-communicable diseases. Since our modern lifestyle plays a crucial role in the etiology of these disorders, lifestyle medicine needs to be an integral part of our strategies to tackle the huge health threats we face today.
